# Association between *MEFV* Mutations M694V and M680I and Behçet’s Disease: A Meta-Analysis

**DOI:** 10.1371/journal.pone.0132704

**Published:** 2015-07-15

**Authors:** Ziyan Wu, Shulan Zhang, Jing Li, Si Chen, Ping Li, Fei Sun, Xiaoting Wen, Wenjie Zheng, Fengchun Zhang, Yongzhe Li

**Affiliations:** Department of Rheumatology and Clinical Immunology, Peking Union Medical College Hospital, Chinese Academy of Medical Sciences & Peking Union Medical College, Key Laboratory of Rheumatology and Clinical Immunology, Ministry of Education, Beijing, China; University of Birmingham, UNITED KINGDOM

## Abstract

**Objective:**

Several studies have identified an association between Behçet’s disease (BD) and mutations in the Mediterranean fever (MEFV) gene, which was originally linked to the autosomal recessive disease, Familial Mediterranean fever. However, no consensus has been reached. Here, a meta-analysis was conducted on published data to comprehensively evaluate this relationship.

**Methods:**

Literature searches were performed in Pubmed, Embase, the Web of Science, and HuGE Navigator databases, in order to identify studies pertaining to the association between *MEFV* mutations and BD. Two investigators independently extracted and evaluated the data from eligible studies. The association between *MEFV* mutations (M694V, M680I, and E148Q) and BD was estimated overall by the odds ratio (OR) and 95% confidence intervals (95% CI). Further analysis was conducted with STATA 12.0 software (Stata Corp.; College Station, TX).

**Results:**

Eligible studies (n=8) included genotyping data obtained from 2538 BD patients and 2792 healthy controls. Of the three mutations, M694V (pooled OR: 2.60, 95% CI: 2.02-3.34) and M680I (pooled OR: 1.74, 95% CI: 1.23-2.46) were found to be associated with BD in the overall analysis. The third mutation, E148Q, however, was not found to be linked with BD (pooled OR: 1.26, 95% CI: 0.69-2.31). Subgroup analysis furthermore revealed that M694V and M680I were risk loci for BD specifically in Turkish patients.

**Conclusions:**

The meta-analysis confirmed that *MEFV* mutations M694V and M680I were associated with BD. Additional studies from other ethnic populations and functional experiments are necessary to determine the extent to which the *MEFV* gene underlies the development of BD.

## Introduction

Behçet’s disease (BD) is a systemic vasculitis characterized by recurrent aphthous ulceration, genital ulcers, ocular inflammation, and skin lesions. The disease has been considered as a seronegative spontaneous arthritis, autoimmune disease, or auto-inflammatory disorder with a probable genetic basis [1]. Familial Mediterranean fever (FMF) is also a genetic autoinflammatory syndrome presenting with episodes of fever, with or without serositis, synovitis, or rashes [2]. Interestingly, certain clinical characteristics are shared by BD and FMF patients, including a unique ethnic prevalence, pathogenesis, and treatment protocols, despite a wide range in clinical manifestations [3,4]. Both BD and FMF, for example, are observed all around the Mediterranean basin, with BD occurring more frequently along the ancient Silk Road, which extended from Asia to the Mediterranean [5,6]. In addition, there are case reports of the co-existence of BD and FMF in individual patients. The intersection of some disease characteristics has therefore led to the proposal that a common genetic susceptibility exists between BD and FMF [7–9].

Specific genes have been associated with BD. So far, HLA-B*51 has been shown to share the strongest association with BD in several different populations [10]. However, genome-wide association studies (GWAS) have also revealed other potential susceptibility loci for BD, such as HLA-A*26, *IL10*, *IL23R-IL12RB2*, *STAT4*, *GIMAP*, *CCR1*, and *KLRC4* [11–15]. Although several GWAS and numerous candidate gene association studies have been conducted to identify susceptibility loci [11–18], they have all been based on the hypothesis-‘common variants and common disease’-which places the emphasis on the identification of common variants [minor allele frequency (MAF) > 5%] rather than rare and/or genetic structure variations. These common variants only explain ~20% heritability, and thus, the existence of a genetic component, the so-called ‘missing heritability’, is indisputable [19].

The Mediterranean fever (MEFV) gene encodes pyrin, a protein of 781 amino acids, which has been associated with the development of FMF. Pyrin is expressed primarily in the myeloid cell lineage, and results in dysregulated inflammation and excessive IL-1β activation [20]. IL-1 is a critical pro-inflammatory cytokine which is also elevated in BD patients [21]. Since the identification of *MEFV* gene mutations in 1997 by the French and the International consortia [22,23], there have been several studies confirming that a high frequency of *MEFV* mutations are also found in BD patients. However, these observations have not been supported by other studies[24–33]. Therefore, a meta-analysis was performed with data from published studies until Jan 22, 2015 in order to comprehensively review the association between *MEFV* mutations and BD.

## Materials and Methods

### Data source

A comprehensive search for *MEFV* mutations and BD was conducted in Pubmed, Embase, Web of Science, and the HuGE Navigator databases up to the date of Jan 22, 2015. And the Pubmed and Embase database were searched by a combination of MeSH, Emtree headings and text words. The keywords used were “Behçet disease”, “Behçet syndrome”, “Adamantiades-Behçet's disease”, “BD”, “Mediterranean fever gene”, “MEFV”, “MEFV mutations”, “MEFV variants”, “MEFV variations”, “M694V”, “M680I”, “E148Q” and so on(S1 Table). No limitations were placed on the type of study performed.

### Inclusion and exclusion criteria

Studies included in the meta-analysis met the following criteria: 1) articles published before Jan 22, 2015; 2) case-control studies evaluating the relationship between *MEFV* mutations and BD; 3) studies with adequate data to allow the estimation of an odds ratio (OR) with 95% confidence intervals (95% CI); 4) studies written in English. Exclusion criteria were the following: 1) case reports or family based studies; 2) studies with no usable data; 3) studies with duplicate data. Authors were contacted in the absence of an electronic full text or available data to calculate the OR and 95%CI.

### Data extraction

In accordance with the inclusion criteria, the following items were extracted independently by two investigators(Si Chen and Ping Li): 1) study characteristics: first author, publication study, title, sample size, study population, genotyping method; 2) number of *MEFV* mutations in BD patients and healthy controls. Any disagreement between the investigators was resolved by discussion until a consensus was reached. If they failed to reach an agreement, a third investigator was consulted to resolve the discrepancies(Yongzhe Li).

### Study quality assessment

Two investigators assessed the quality of the included studies independently with the Newcastle Ottawa Scale (NOS). Three items were included in the NOS: selection (0–4 points), comparability (0–2 points), and outcome (0–3 points). NOS scores > 6 were considered high quality [34].

### Statistical methods

Meta-analysis was conducted with STATA 12.0 software (Stata Corp.; College Station, TX, USA). The association between *MEFV* mutations and BD was estimated by pooled OR and 95% CI. Between-study heterogeneity was assessed with the Q-test (*p*-value [*p*_het_] < 0.10 was regarded as statistically significant heterogeneity) and I^2^ statistics (75 ≤ I^2^ < 100 = extreme heterogeneity, 50 ≤ I^2^ < 75 = high heterogeneity, 25 ≤ I^2^ < 50 = moderate heterogeneity, I^2^ < 25 = no heterogeneity). A random-effects model was applied unless there was no significant between-study heterogeneity, in which case a fixed-effects model was used [35]. Potential publication bias was estimated with the Begg’s funnel plot and the Egger’s test where the threshold *p*-value was set at 0.10 [36,37]. Sensitivity analysis was performed to assess the influence of each study on the pooled OR. All *p*-values < 0.05 were considered statistically significant. Power analysis was performed using the software—PS: Power and Sample Size Calculation [38].

## Results

### Study selection and characteristics

**Fig 1:**
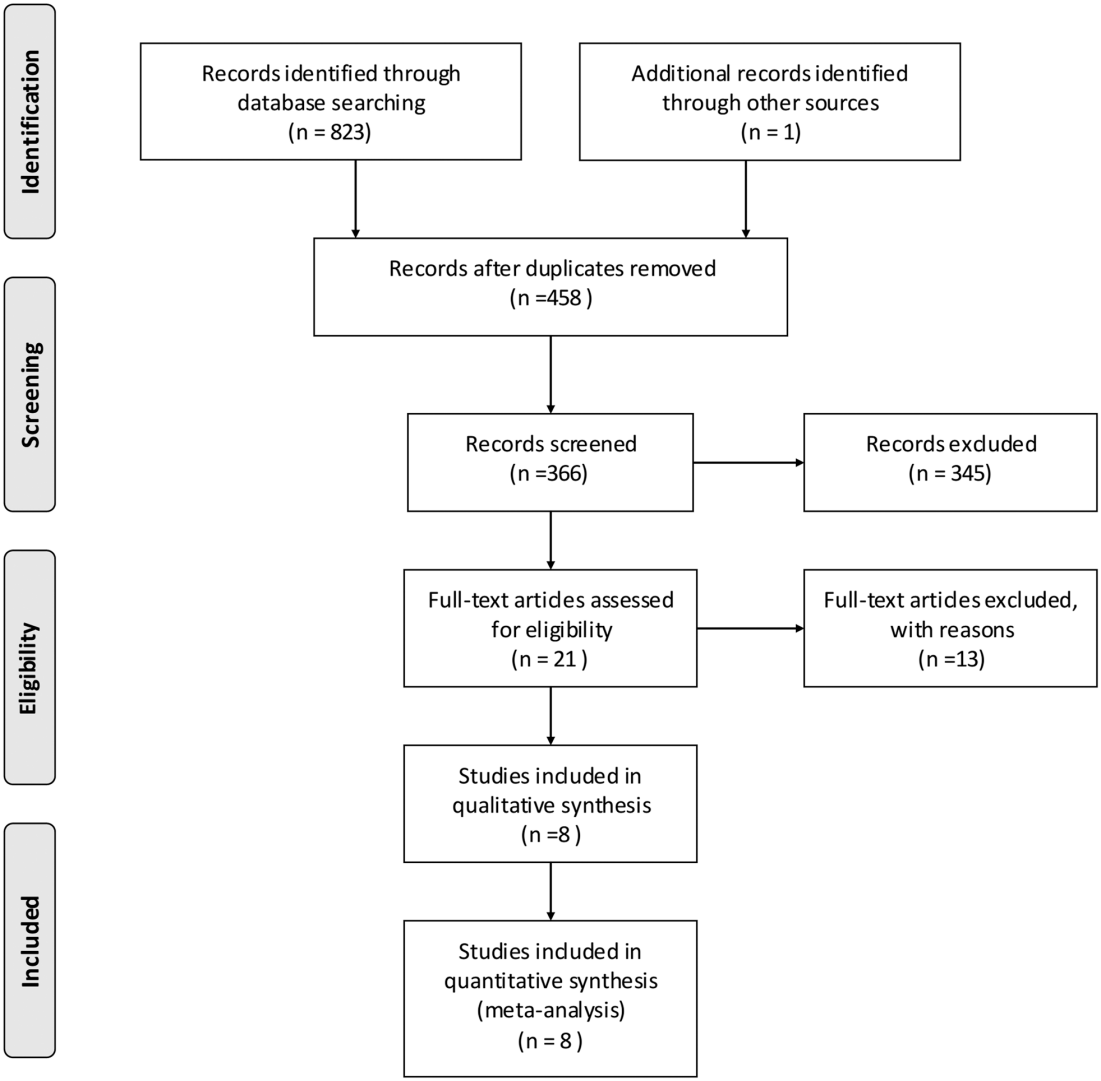
Flowchart for the selection of the eight studies in the meta-analysis.

A search of the databases yielded 823 potential articles related to *MEFV* mutations and BD (Pubmed: 155, Embase: 341, Web of Science: 316 and HuGE Navigator:11). The PRISMA checklist was used as a guide to identify eligible studies for the meta-analysis (S1 File). After the removal of duplicates and irrelevant articles, 21 full-text articles were retrieved for further details. An additional two duplicated publications were excluded as well as nine studies which did not satisfy the inclusion criteria and two more studies with no usable data. Finally, eight studies performed with a total of 2538 BD patients and 2792 healthy controls were included in the meta-analysis (Fig 1).

The baseline characteristics of these eight studies are listed in Table 1, All the included articles were published in English, and five were conducted on Turkish cohorts. The NOS scores for these eight studies were the following: 8 (n = 2), 7 (n = 5), 6 (n = 1), and 5 (n = 1).

**Table 1:** Characteristics of included studies.

Author	Year	Gene	Mutations	Population	No.of case	No.of control	Genotyped method	Scores of NOS	Power(%)*	Ref
Touitou, I.	2000	MEFV	M694V	Mixed	57	114	PCR-RFLP	7	46.7	24
			E148Q						13.3	
Imirzalioglu, N.	2005	MEFV	M694V	Turkish	42	66	PRONTO FMF Basic Kit	7	65.7	26
			M680I						4.9	
			E148Q						61.6	
Espinosa, G.	2005	MEFV	E148Q	Spanish	50	100	ABI Sequencing	7	8.6	33
Esmaeili, M.	2011	MEFV	M694V	Turkish	53	200	PCR-RFLP	7	77.0	28
			M680I						65.7	
			E148Q						5.0	
Yazici, A.	2012	MEFV	M694V	Turkish	100	100	PCR reverse hybridization method	7	18.0	29
			M680I						27.4	
			E148Q						61.1	
Konstantopoulos,K.	2012	MEFV	M694V	Greek	96	140	Non-isotopic RNase cleavage assay	5	5.1	30
			M680I						5.1	
Tasliyurt, T.	2013	MEFV	M694V	Turkish	207	200	PCR-RFLP	6	17.8	31
			M680I						55.8	
			E148Q						93.3	
Kirino, Y.	2013	MEFV	M694V	Turkish	1933	1872	Sequenom MassArray,Sanger sequencing	8	100.0	32
			M680I						54.0	
			E148Q						12.5	

PCR-RFLP: Polymerase Chain Reaction-Restriction Fragment Length Polymorphisms;

*Power analysis was performed using the software

PS: Power and Sample Size Calculation

### MEFV mutations and BD

#### M694V

**Fig 2:**
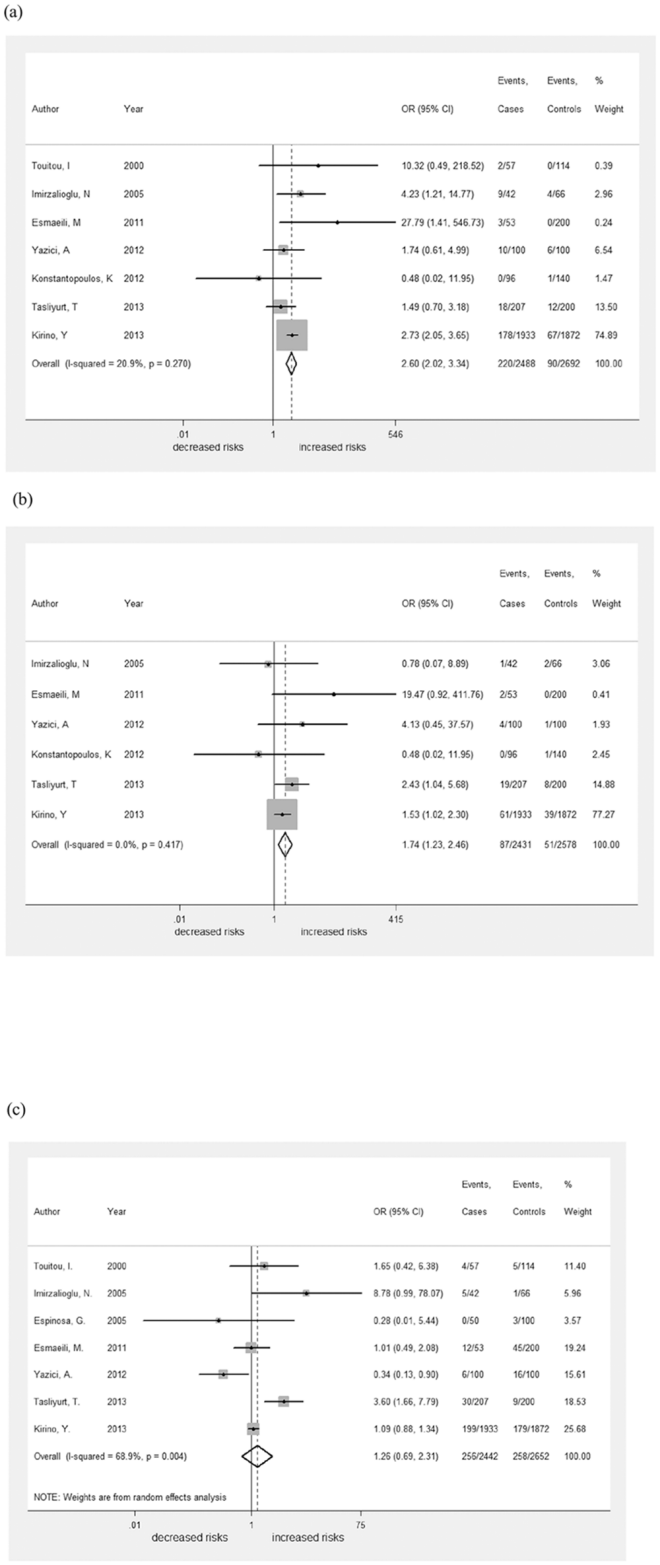
Forest plot of the association of individual mutations with BD. A fixed-effects model for the OR with 95% CI was used to detect an association between M694V and M680I mutations with BD, whereas a random-effects model for the OR with 95% CI was used to estimate the association of the E148Q mutation with BD. M694V (pooled OR: 2.60, 95% CI: 2.02–3.34) and M680I (pooled OR: 1.74, 95% CI: 1.23–2.46) were found to be associated with BD in the overall analysis. E148Q, however, was not found to be linked with BD (pooled OR: 1.26, 95% CI: 0.69–2.31).

Seven of the studies, including 2488 BD patients and 2692 controls, evaluated the association between the *MEFV* mutation M694V and BD. All studies, with the exception of one based on a Greek cohort, revealed a higher frequency of the M694V mutation in BD patients (220/2488; 8.84%) relative to unaffected controls (90/2692; 3.34%). To demonstrate the association between M694V and BD, a fixed-effects model was used, there was no significant between-study heterogeneity (I^2^ = 20.9%, *p*_het_ = 0.270). The overall analysis revealed that M694V was associated with BD (pooled OR: 2.60, 95% CI: 2.02–3.34, Fig 2). Subgroup analysis also demonstrated that M694V was associated with BD in Turkish patients (pooled OR: 2.60, 95% CI: 2.02–3.35, S1 Fig).

#### M680I

The M680I mutation was investigated for association with BD in six studies which were included in the meta-analysis. M680I occurred at a slightly higher frequency in BD patients (87/2431; 3.58%) relative to unaffected controls (51/2578; 1.98%). A fixed-effects model was adapted to calculate the overall OR and 95% CI, as I^2^ was 0.7% and *p*_het_ was 0.411. In this model, M680I was also found to be associated with BD (pooled OR: 1.74, 95% CI: 1.23–2.46, Fig 2). Again, the disease was found to be linked to M680I in Turkish patients through subgroup analysis (pooled OR: 1.77, 95% CI: 1.25–2.52, S1 Fig).

#### E148Q

A third mutation, E148Q, was also included in the meta-analysis. Data for patients and controls harboring the mutation were extracted from seven studies. The mutation occurred in 256/2442 BD patients (10.48%) and 258/2652 controls (9.73%). The between-study heterogeneity was significant (I^2^ = 68.9%, *p*_het_ = 0.004), so that a random-effects model was conducted to estimate the overall effects (pooled OR: 1.26, 95% CI: 0.69–2.31, Fig 2). However, no significant association between the E148Q variant and BD was identified in a subgroup analysis of Turkish patients (pooled OR: 1.31, 95% CI: 0.65–2.64, S1 Fig).

### Publication bias and sensitivity analyses

**Fig 3:**
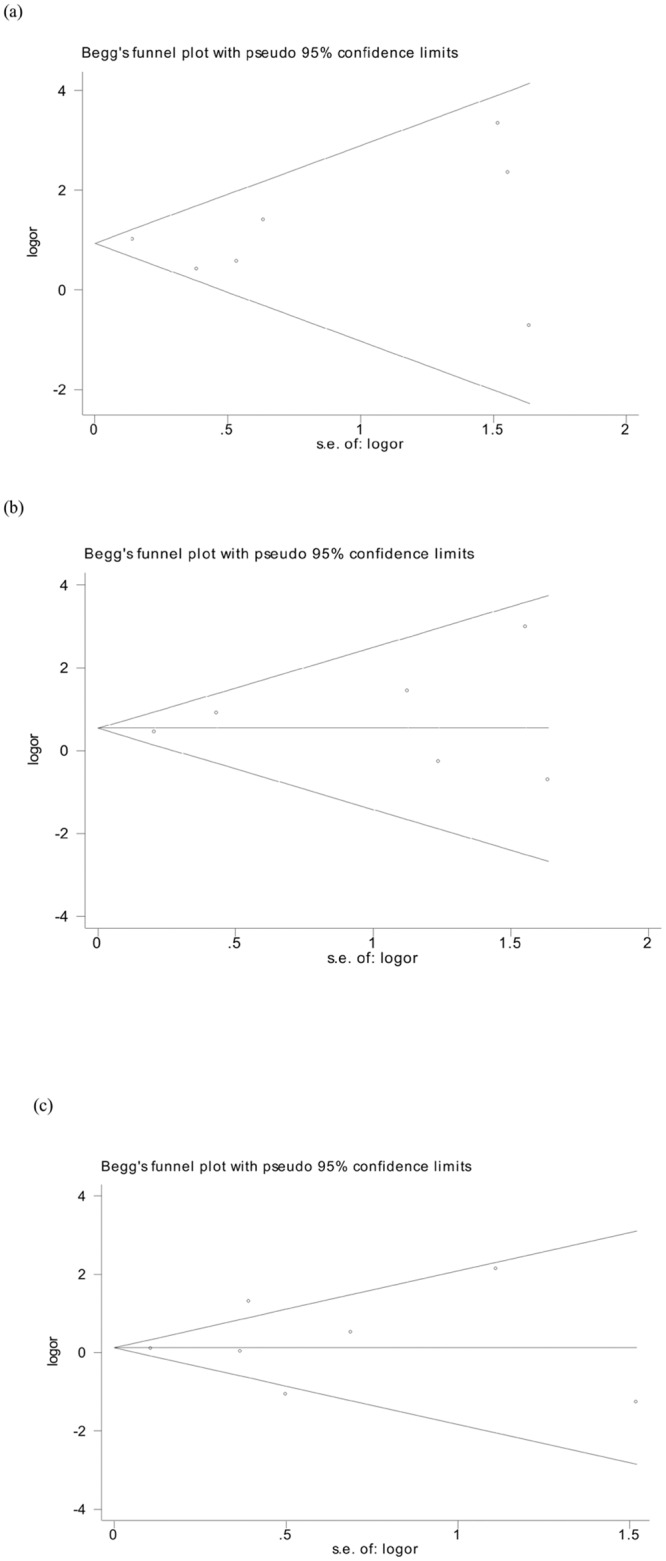
Publication bias was not evident in the studies used to determine the relationship between *MEFV* mutations and BD. Symmetry in Begg’s funnel plots demonstrate the absence of publication bias in the studies investigating the association of (A) M694V, (B) M680I, and (C) E148Q mutations with BD.

**Fig 4:**
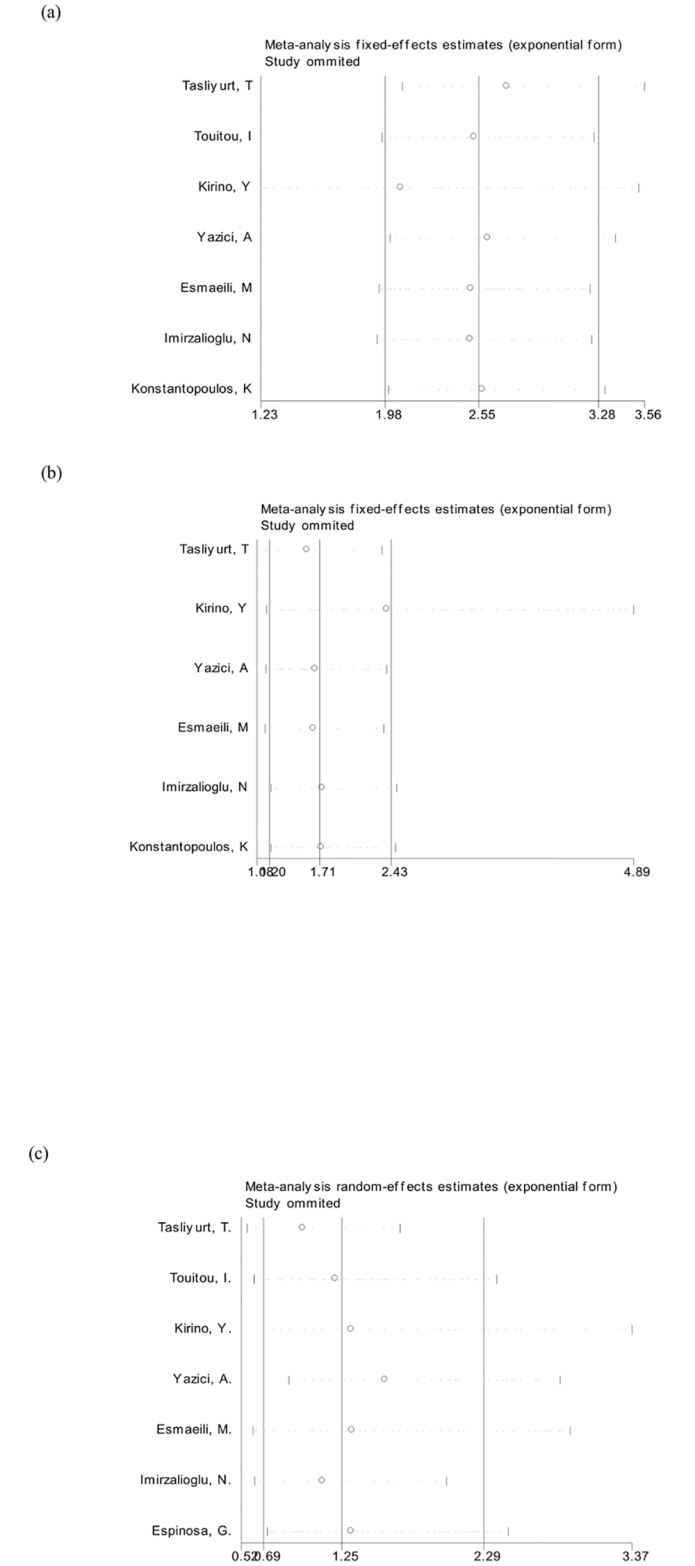
Sensitivity analysis demonstrates that no single study dominated the results. The summary odds ratio coefficients for the association of (A) M694V, (B) M680I, and (C) E148Q mutations with BD illustrate that statistically similar results were obtained after sequentially excluding each study, confirming the stability of the meta-analysis.

A Begg’s funnel plot was used to reveal any publication bias influencing the analysis. The funnel plot did not show significant sign of asymmetry for M694V, M680I and E148Q (Fig 3). The Egger’s test was also conducted and indicated an absence of publication bias (t = 0.21,0.68,0.34; p = 0.84,0.54,0.75, respectively). Furthermore, statistically similar results were obtained after sequentially excluding each study, confirming the stability of the meta-analysis, and indicating that the results were stable and reliable (Fig 4).

## Discussion

In the absence of a clear mechanism for the development of the disease, the fundamental nature of BD remains controversial: is it an autoimmune or auto-inflammatory disease [39]? In classic autoimmune diseases, such as systemic lupus erythematosus (SLE), specific auto-antibodies, anti-dsDNA or anti-Sm, are used to diagnose the disease [40]. BD, however, remains undefined in terms of a specific diagnostic auto-antibody. Therefore, based on the fact that inflammation and anti-inflammatory processes play a prominent role in the initiation and development of BD [41,42], it could be classified as an auto-inflammatory disease. In this respect, BD has similarities to FMF which is also an auto-inflammatory disease characterized by acute episodes of fever, abdominal pain, chest pain, arthritis, or erysipelas-like skin eruptions. In fact, it is sometimes difficult to clearly distinguish between the two inflammatory diseases, as the main symptoms are not highly specific [3,4]. Furthermore, FMF is prevalent in the Middle East, mainly affecting Turkish, Armenian, Arabian, and Sephardic Jewish patients, which is also the epidemic area for BD [6]. FMF is autosomal-recessive [2], and *MEFV* mutations underlie the disease in more than 50% of patients [43]. MEFV mutations have therefore become the focus of studies on BD as a potential genetic component in development of the disease [24–33]. In addition, IL23R, ERAP1 and IL10 were associated with both BD and inflammatory disease, which suggested that BD and auto-inflammatory disease may have some shared genes or inflammatory pathways[44]. Our meta-analysis, which included all available studies and significantly increased the power for detecting association, is the first to investigate the relationship between *MEFV* mutations and BD. M694V and M680I, two high penetrance mutations, were associated with BD, whereas E148Q, a low-penetrance pyrin mutation, was not linked to the disease.

Meta-analysis enables investigators to resolve discrepancies raised by individual studies. In most individual studies, a high frequency of *MEFV* variants has been observed in BD relative to healthy controls [24–29,31,32], but studies performed on Greek and Spanish populations differ [30,33]. The most recent study demonstrated, in fact, that the frequency of *MEFV* mutations in BD patients was 39.13%, while that for unaffected controls was 19.00% [31]. One potential source of disagreement among individual studies was heterogeneity, which may be attributed to different study populations, variation in sample sizes or genotyping methods, or an underlying diversity in the patient population in each study. The power analysis revealed that 655 cases and 655 healthy controls would have more than 80% power (α = 0.05) for detecting association with an OR of 1.87 for disease susceptibility in cases relative to controls, based on 0.05 for the mutation rate in controls, the average rate of the three tested SNPs in our studies[45]. Based on our statistical analyses, M694V and M680I did not exhibit significant heterogeneity between studies. However, the association of E148Q with BD was less clear based on individual studies as only a single study of small sample size observed a relationship between the variant and BD [31], whereas the other six studies all failed to investigate E148Q as a potential susceptibility locus [24–26,28,29,32,33].

The *MEFV* gene (14600 bp) is located on chromosome 16p.13.3 and consists of ten exons. More than 300 variations in *MEFV*, with M694V, M680I, and E148Q as the most common, have been identified[46]. MEFV mutations are very common in several Mediterranean populations,but it is not the same mutation when it expand in other population, which reinforces the possibility of a selective advantage. For instance: M694V and M680I are located in exon 10 and more specific in several Mediterranean populations, while E148Q in exon2 is frequently encountered in the general population (up to 30% in the Asian population according to the Ensembl database) [43]. In addition to their association with BD, these mutations have been linked with different clinical manifestations of the disease[25]. For example, M694V has been associated with ankylosing spondylitis and inflammatory bowel disease [47,48]. M694V is also a risk allele for more severe inflammatory manifestations of FMF, including an increased risk for amyloid deposition [49]. Therefore, the result that M694V emerged as a susceptibility locus for BD in our meta-analysis potentially implicates this variant as the basis for some specific inflammatory processes. Pyrin, which is the protein product of *MEFV*, is composed of at least of five domains (PYD, bZIP, B-box, coiled-coil and B30.2/SPRY) [20]. The B30.2/SPRY domain, at the C terminus of the protein, is encoded by exon 10 which clusters most severe MEFV mutations. The B30.2 domain interacts with caspase-1 and that this regulates the production of mature IL-1β. In addition, pryin increases caspase-1 activity and induces IL-1β expression in a dose-dependent manner. PYD domain at the N-terminal of pyrin, could activate NF-ĸB by increasing degradation of IĸB-α[50,51]. Previous studies had found decreased expression of MEFV mRNA in peripheral blood leukocytes obtained from FMF patients[52]. And there was an inverse correlation between MEFV mRNA expression and FMF clinical severity score. Also, MEFV mRNA expression was related to the type of mutation. M694V was associated with the lowest MEFV mRNA levels, while E148Q was associated with the highest. In addition, there was a dose-dependent relationship between the number of mutations and MEFV transcripts[53]. Monocytes from FMF patients exhibited growing IL-1β secretion, which correlated with the number and penetrance of MEFV mutations[54]. In a mouse model for severe auto-inflammation, *MEFV* variants (M694V and M680I) were proposed to be gain-of-function mutations which lead to the activation of caspase-1 and IL-1β, two proteins with established roles in inflammation [55].

There are several caveats, however, to the meta-analysis. Firstly, publication bias, due to the tendency to publish studies with positive results, is a well-established and inherent problem when interpreting the results of meta-analyses. Although no significant publication bias for individual mutations was apparent in this study, a selective reporting bias could also affect the results despite evaluation at multiple genetic markers if only selected subgroups with the most significant results are reported [56]. Secondly, the quality of the included studies influences the reliability of the meta-analysis. The NOS scores to estimate the quality of each study ranged from 5 to 8 indicating that the studies were of sufficient quality. Thirdly, the study was performed by combining the results of retrospective case-control studies. Finally, most of the studies were conducted on Turkish patients, and therefore, although the results of the study are highly provocative, they must be interpreted carefully.

In summary, the meta-analysis further established a potential role for variants of the *MEFV* gene, M694V and M680I, in the pathogenesis of BD in Turkish patients. However, additional studies from more diverse ethnic populations and functional experiments are necessary in order to confirm and to extend these observations.

## Supporting Information

S1 Fig Subgroup analysis reveals an association of M694V and M680I with BD in Turkish patients. (TIF)

S1 File PRISMA checklist. (DOC)

S1 Table The searching strategy used in Pubmed and Embase database. (DOC)
