# Supplementary figures and images for: Association between MEFV Mutations M694V and M680I and Behçet’s Disease: A Meta-Analysis
Source: PLoS One. 2015 Jul 15;10(7):e0132704. doi: 10.1371/journal.pone.0132704 (PMC4503748; doi:10.1371/journal.pone.0132704)

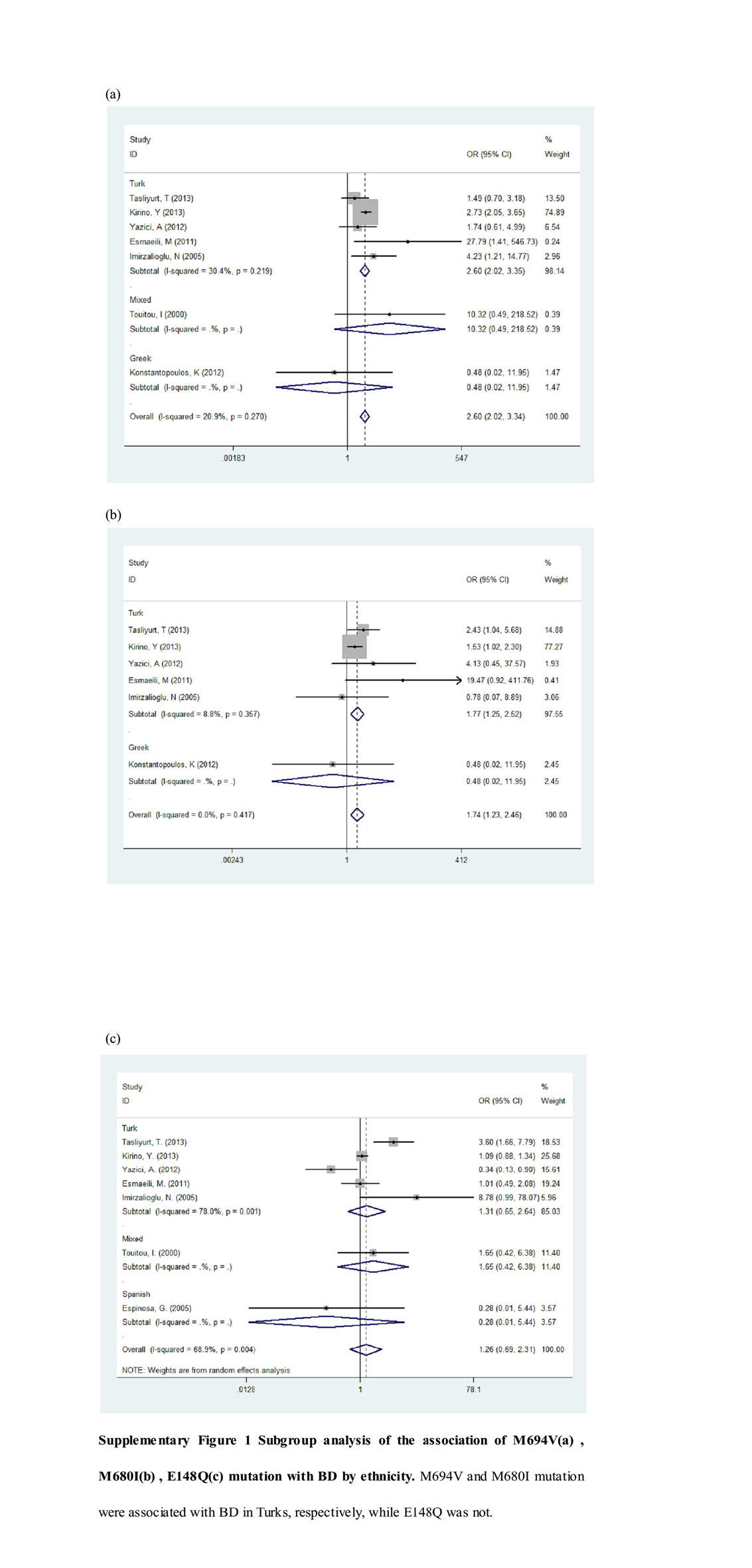

Supplement: S1 Fig — (TIF) [file pone.0132704.s001.tif]
