# Supplementary material for: Association between MEFV Mutations M694V and M680I and Behçet’s Disease: A Meta-Analysis
Source: PLoS One. 2015 Jul 15;10(7):e0132704. doi: 10.1371/journal.pone.0132704 (PMC4503748; doi:10.1371/journal.pone.0132704)
Supplement: S1 Table — (DOC) [file pone.0132704.s003.doc]

**Supplementary Table S1: the searching strategy used in Pubmed and Embase database**

| Database | Behçet's Disease | MEFV |
| --- | --- | --- |
| Medline MeSH Headings | 1. Behcet Syndrome / 2. Retinal Vasculitis/ | 1. Familial Mediterranean Fever / 2. marenostrin / |
| Embase Emtree headings | 1. 'behcet disease'/exp 2. 'retina vasculitis'/exp | - 1. 'familial mediterranean fever'/exp |
| Text words (used in both databases) | 1. Behcet Disease.tw  2. Behcet Triple Symptom  Complex.tw  3. Triple-Symptom Complex.tw  4. Behçet Disease.tw  5. Old Silk Route Disease.tw  6. Triple Symptom Complex.tw  7. Adamantiades-Behcet Disease.tw  8. Behcet's Syndrome.tw  9. Behcets Syndrome.tw  10. BD.tw  11. BS.tw | 1. mefv.tw 2. Familial Mediterranean Fever.tw 3. Mediterranean fever gene.tw 4. MEFV mutations.tw 5. MEFV mutation.tw 6. MEFV variants.tw 7. MEFV variations.tw 8. Mediterranean fever gene mutations.tw 9. Mediterranean fever gene mutation.tw 10. Mediterranean fever gene variants.tw 11. Mediterranean fever gene variations.tw 12. M694V OR rs61752717.tw 13. M680I OR rs28940580.tw 14. E148Q OR rs3743930.tw |
